# Supplementary figures and images for: WTools: A MATLAB-based toolbox for time-frequency analysis of infant data
Source: PLoS One. 2025 May 7;20(5):e0323179. doi: 10.1371/journal.pone.0323179 (PMC12058173; doi:10.1371/journal.pone.0323179)

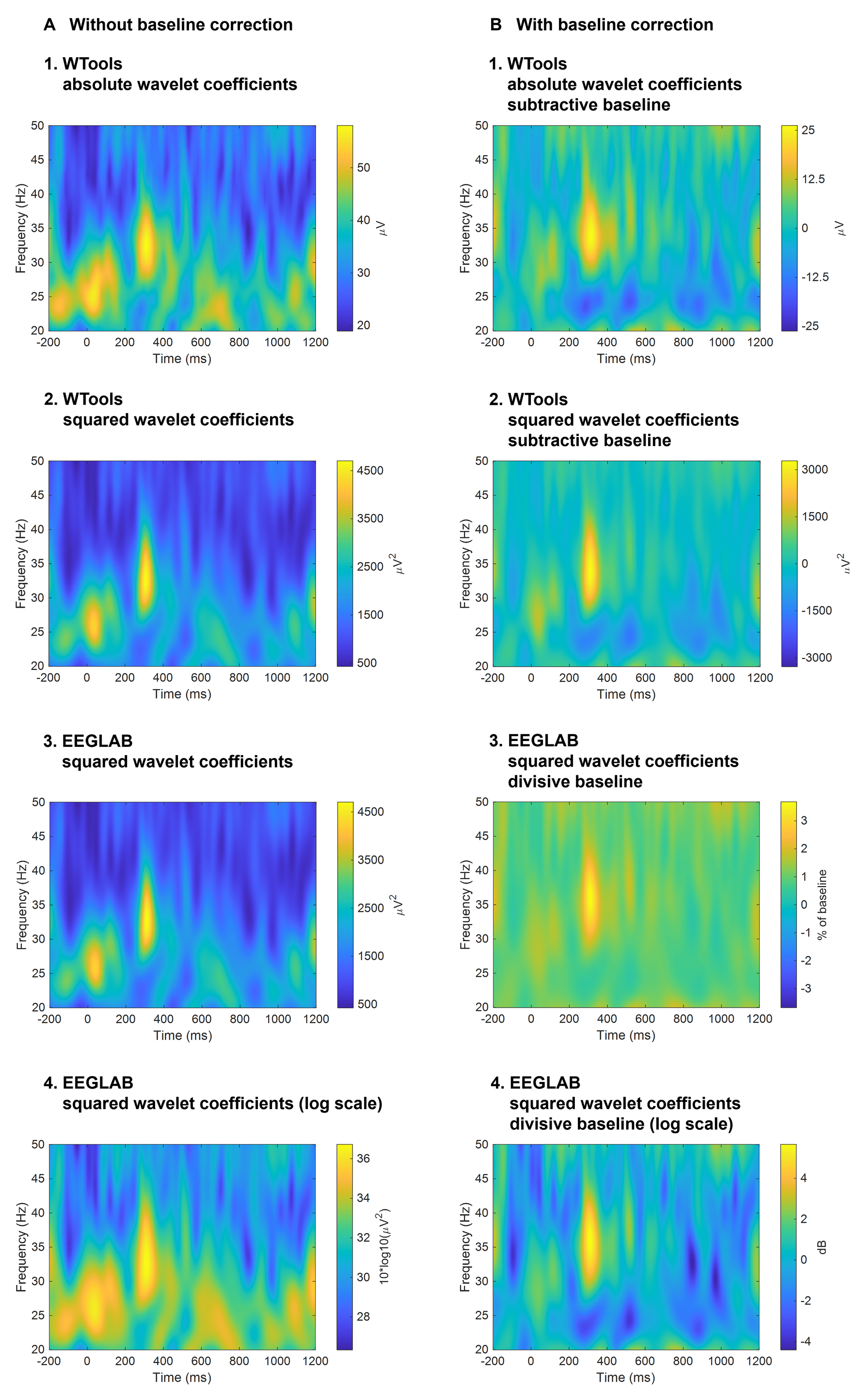

Supplement: S1 Fig — We show a comprehensive overview of results under all analyses pipelines, which derived from the orthogonal crossing of the manipulated settings (i.e., baseline correction, complex wavelet manipulation, log-transformation). We ran four time-frequency analyses in WTools corresponding to the orthogonal crossing of 1) baseline correction (with or without subtractive baseline) and 2) complex coefficient transformation (absolute or squared value). We ran four time-frequency analyses in EEGLAB corresponding to the orthogonal crossing of 1) baseline correction (with or without divisive baseline) and 2) log-transformation of the time-frequency results to convert them to the standard dB scale (with or without log-transformation). For all time-frequency analyses, we applied the following core parameters: frequency range = [10, 90] Hz; time window = [-200, 1200] ms; number of wavelet cycles = 7. Time-frequency plots correspond to one representative participant (“04”), channel (“22”, corresponding to Fp2) and condition (“DG”). (TIF) [file pone.0323179.s001.tif]

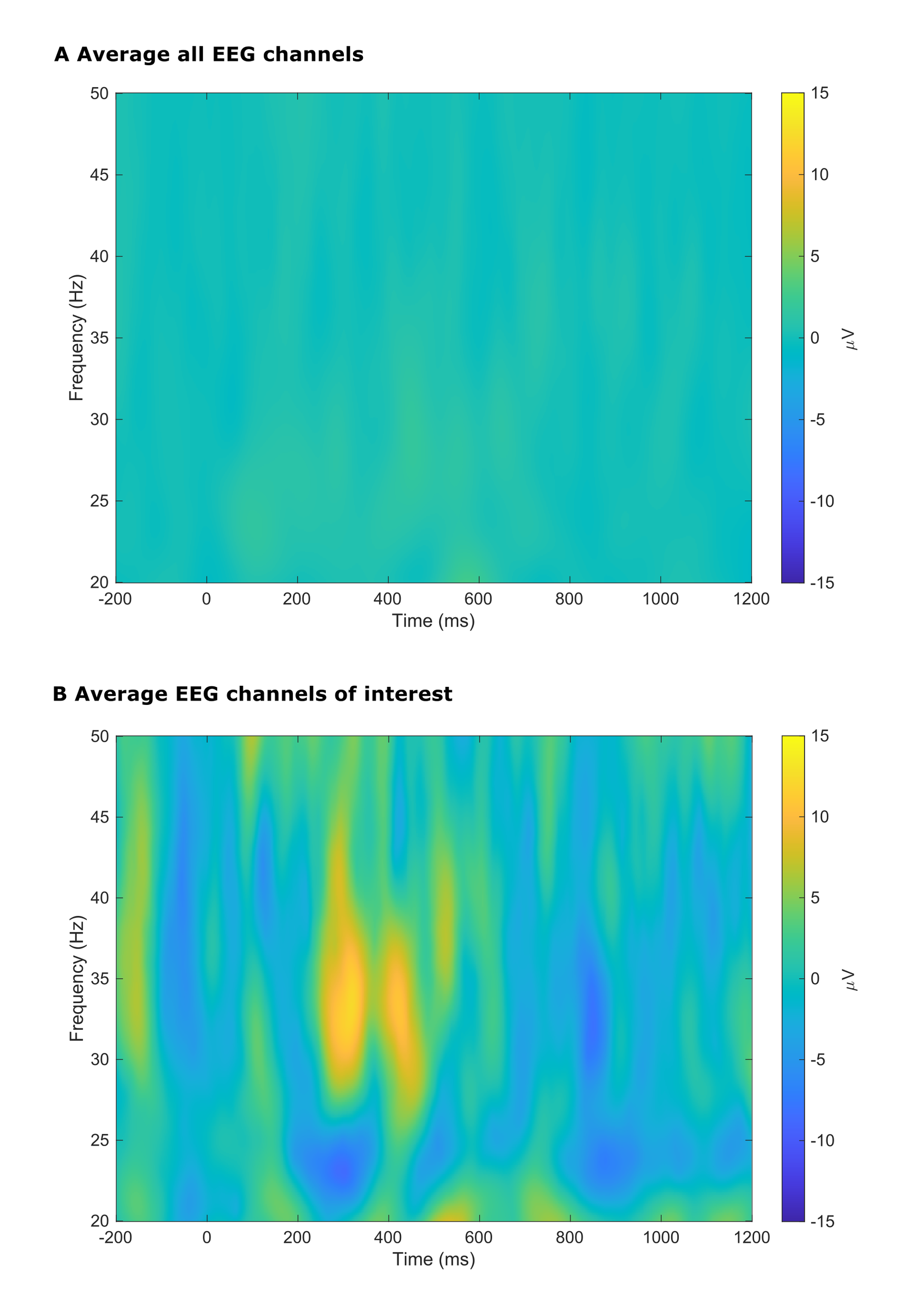

Supplement: S2 Fig — Time-frequency plots averaged over all EEG channels (A) compared to averaging over a desired subset of EEG channels (B). These are the same three frontal channels of interest (“9”, “15”, and “22”, corresponding to Fp1, Fpz, and Fp2, respectively) used in the original publication from which the empirical data were taken [1]. Results were obtained using the “Channels Average” functionality for one representative participant (“04”) and condition (“DG”), under the default WTools pipeline. (TIF) [file pone.0323179.s002.tif]
